# Integrating Hyperthermia into Modern Radiation Oncology: What Evidence Is Necessary?

**DOI:** 10.3389/fonc.2017.00132

**Published:** 2017-06-30

**Authors:** Jan C. Peeken, Peter Vaupel, Stephanie E. Combs

**Affiliations:** ^1^Department of Radiation Oncology, Klinikum rechts der Isar, Technische Universität München, München, Germany; ^2^Department of Radiation Sciences (DRS), Institute of Innovative Radiotherapy (iRT), Helmholtz Zentrum München, Neuherberg, Germany

**Keywords:** hyperthermia, radiation oncology, reirradiation, infrared-A, thermoradiotherapy

## Abstract

Hyperthermia (HT) is one of the hot topics that have been discussed over decades. However, it never made its way into primetime. The basic biological rationale of heat to enhance the effect of radiation, chemotherapeutic agents, and immunotherapy is evident. Preclinical work has confirmed this effect. HT may trigger changes in perfusion and oxygenation as well as inhibition of DNA repair mechanisms. Moreover, there is evidence for immune stimulation and the induction of systemic immune responses. Despite the increasing number of solid clinical studies, only few centers have included this adjuvant treatment into their repertoire. Over the years, abundant prospective and randomized clinical data have emerged demonstrating a clear benefit of combined HT and radiotherapy for multiple entities such as superficial breast cancer recurrences, cervix carcinoma, or cancers of the head and neck. Regarding less investigated indications, the existing data are promising and more clinical trials are currently recruiting patients. How do we proceed from here? Preclinical evidence is present. Multiple indications benefit from additional HT in the clinical setting. This article summarizes the present evidence and develops ideas for future research.

## Introduction

Hyperthermia (HT) is defined as an exogenous, supraphysiological elevation of tissue/body temperature. The beginning of modern HT dates back to the 1700s when remissions of malignant tumors were repeatedly associated with concomitant bacterial infections. This effect was first systematically investigated at the break of the 19th century by Coley (1). Patients with unresectable sarcomas received injections of bacterial vaccines for fever induction. In total, a cure rate of 20% was achieved (2). It took several decades of technological developments for local/locoregional heat application until HT alone became available for clinical application.

Nowadays, HT is either administered independently or, more often, in combination with radiotherapy (RT) or chemotherapy (CT). HT alone is being used for direct ablation of single tumor lesions with temperatures exceeding 50°C. Multiple techniques are being used to obtain necessary temperature coverage such as high-intensity focused ultrasound and radiofrequency-, microwave-, or infrared laser-based heating *via* ablation catheters directly inserted into the tumor (3).

In bimodal treatment schemes such as thermoradiotherapy (RTHT) and chemoradiotherapy (RTCT) as well as in trimodal thermochemoradiotherapy (RTHTCT), HT is utilized for augmentation of treatment effects of the concomitant oncological therapy. Necessary tissue temperatures are significantly lower ranging from 39 to 43°C (4, 5).

In this literature-based review, a brief introduction to HT physiology, cell biology, and immune response is given to examine the underlying modes of action of HT. Currently used HT techniques for heat delivery and temperature control are described. The clinical evidence of combining RT with HT is summarized and sorted per tumor entity. To this end, a PubMed search was conducted searching for the term “hyperthermia” in combination with tumor entities treatable by RT, and terms describing technical aspects such as “biology,” “physiology,” “chemotherapy,” and “radiation therapy.” Special emphasis is given to recent meta-analyses and published prospective trials.

## Preclinical Evidence

### Changes in Perfusion and Oxygenation

Data and the respective interpretation of HT-induced changes in perfusion and oxygenation remain controversial and are briefly described in the following. A comprehensive review of this topic has been published by Vaupel and Kelleher (6). There is evidence that mild HT can increase blood perfusion of the heated tissue, preferentially at the beginning of tumor heating (7, 8). It has been reported that this can lead to increased oxygen delivery *via* an improvement of microcirculation (9). This is especially true in cases when the oxygen demand of the tissue is reduced. It has been proposed that direct heat-dependent cell killing and loss of mitochondrial membrane potential contribute to this phenomenon (10, 11). On the contrary, other studies showed increased oxygen consumption at elevated tissue temperature (van’t Hoff’s law!) counteracting the oxygenating effect of increased perfusion (12). An increase in oxygen availability may favor oxygenation of hypoxic cells (7). The effect appears to be preferentially in diffusion-limited, chronic hypoxia (13, 14). Whether the radiosensitizing effect outlasts the time frame of increased perfusion remains so far unclear. Some studies have reported increased perfusion extending over 24 h after HT, which would benefit following RT/CT sessions (15, 16). Other studies could not reproduce this result (17). As hypoxia is a central causative factor for radioresistance, a decrease in hypoxia by HT may be responsible for the observed radiosensitization.

### Induction of Cell Death

Hyperthermia has been shown to confer cell death by apoptosis or mitotic catastrophe (18, 19). It has been reported that HT triggers unfolding of especially heat-labile non-histone nuclear proteins leading to aggregation, due to exposition of hydrophobic groups, with surrounding proteins and subsequent association with the nuclear matrix. As consequence, basic nuclear matrix-dependent functions such as transcription, replication, or DNA repair are impaired (20, 21). Malfunction of DNA replication finally causes chromosome aberrations, genome instability, and cell death by mitotic catastrophe (22). Apoptosis may be mediated by cell death membrane receptor activation and subsequent caspase 3 activation (23). The extent of apoptosis appears to differ among different tumor types (24). In addition, the permeability of the cellular and mitochondrial membranes is altered leading to cellular Ca^2+^-spikes as well as mitochondrial depolarization with resulting bursts of reactive oxygen species. Both mechanisms may further enhance protein instability and apoptosis (25–27).

### Inhibition of DNA Repair Mechanisms

As mentioned above, there is sufficient evidence showing inhibition of DNA repair mechanisms upon HT. Krawczyk et al. have demonstrated inhibition of homologous recombination at clinically achievable mild HT temperatures (41–42.5°C) associated with BRCA2 degradation and its reduced accumulation at double-strand break sites (28). Further on, HT impairs the function of the Ku heterodimer by reducing its DNA-binding capacity and preventing the initiation of non-homologous end joining at DNA double-strand breaks sites (29). In addition, base excision after cell radiation has been shown to be reduced upon heat administration (30). In summary, HT acts on multiple levels including excision repair, non-homologous end joining, and homologous recombination influencing the repair of DNA lesions as well as single-strand and double-strand breaks (29–31). As a consequence, the effects of DNA damaging treatments such as CT or RT are enhanced. A more detailed review was recently published discussing existing evidence (32).

### Immune Stimulation

Besides direct effects on cell metabolism, HT appears to trigger multiple immune responses on local and systemic levels. Toraya-Brown and Fiering published a thorough review covering this aspect (33). In summary, HT increases expression of immunogenic surface receptors such as MICA and MHC-I enhancing effectiveness and function of natural killer (NK) cells and of CD8^+^ cells, respectively (34, 35). The expression of heat shock proteins (HSPs) such as HSP70 is increased. After binding intracellular proteins, HSPs get secreted stimulating the activity of NK cell- and antigen-presenting dendritic cells (36, 37). Presentation of these tumor antigens can cause specific antitumor immune responses effected by CD8^+^ cells (38). Tumor antigens are also provided by increased release of exosomes (39). Direct enhancement of immunogenic activity of leukocytes is mediated by increased lysis acitivy of NK cells, activation of macrophages, maturation of dendritic cells, and increased IFNy production as well as cytotoxicity of CD8^+^ cells (34, 40–42). In addition, immune cell trafficking is enhanced by increased perfusion and permeability (43). Following elevated intratumoral IL-6 signaling, it may further be facilitated by increased cell adhesion molecule expression such as ICAM-I (44).

## Heat Delivery and Temperature Control

### Heating Techniques

Heating techniques can be divided by the size, penetration depth, and region of energy deposition. Local or regional HT is mostly used to enhance local therapy such as RT or CT. Alternatively, hyperthermic isolated limb perfusion to administer CT agents is performed. Whole-body hyperthermia (WBHT) has been applied either alone or in combination with CT for the treatment of metastatic disease. Different approaches including capacitive, radiative, infrared-A, or ultrasound have been used for clinical HT treatments (45). The clinically most relevant methods are described in the following.

#### Capacitive Heating Systems

Capacitive heating systems work with two electrodes positioned on both sites of the body with direct body contact using a water bolus. Heat is induced by the resulting currents and is directed toward the smallest electrode (46). Capacitive heating tends to create high power densities around the bolus’ edges but good heat coverage of targets inside of the fat layer (47). On the contrary, in obese patients, therapy-limiting local hot spots can occur causing painful subcutaneous burns (48).

#### Radiative Heating Systems

Radiative heating systems work with frequencies ranging from 75 to 915 MHz (spectrum of radiowaves and microwaves) and use a water bolus for electromagnetic coupling. Compared to capacitive heating, radiative systems appear to yield better power disposition and temperature distribution leading to better target coverage (47). The applicable temperature is sometimes limited due to local temperature hot spots. The accuracy of such systems depends on construction details such as the number, positioning and design of antennas or properties of the water bolus (49). In recent years, increasingly complex systems have been introduced comprising multiple antennas such as the commercially available Sigma applicators, build in a circular arrangement, or the AMC-8 phased array HT system (50, 51). Site-specific systems such as the HYPERcollar3D system, which was developed for the treatment of carcinomas of the head and neck, take into account local characteristics of target areas to optimize temperature coverage (52). Alternatively, antennas can be interstitially implanted or used for endocavitary HT in direct approximation to tumors (45). In order to perform superficial or interstitial HT, heating systems are used applying higher frequencies such as 915 MHz (53).

#### Walter-Filtered Infrared-A (wIRA)-Based Systems

Walter-filtered infrared-A-based systems have successfully been used for the treatment of superficial tumors (54, 55). Infrared-A radiation is generated by a halogen lamp, passing through a water filter. The range of therapeutically relevant temperatures is limited to a depth of 15–20 mm [with good therapeutic temperature coverage (56)]. Due to the technical setup a very short interval between HT and RT combined with heat isolation procedures enables quasi-simultaneous RTHT, optimizing the synergistic effect (see below).

Pretherapeutic hyperthermia treatment planning can be used to optimize tumor temperatures. It uses dielectric models created on the basis of segmented CT or MRI data assigning literature-based dielectric properties to distinct tissue types. So, the specific absorption rate (SAR) of the respective tissue can be calculated and used for a finite element-based prediction of temperature distributions. In clinical studies, calculated SAR values correlated well with measured SAR values, relative temperature increase and clinical data regarding hot spots in patients with pelvic tumors (57–59).

### Treatment Temperatures

Preclinical experiments were initially performed with relatively high temperatures ranging from 43 to 45°C focusing on direct cytotoxic effects. However, in the clinical setting, temperatures above 42.5°C were only achieved in small tumor subvolumes. While trying to reach targeted temperatures, therapy-limiting hotspots occurred causing substantial side effects. In these cases, this led to a reduction of target temperatures or early termination of HT (60, 61). This was regarded as failure of delivering adequate thermal doses, which lead to a rapid decline in HT-usage in the mid-to-late 1990s. It took several more years until the beneficial effects of mild HT (39.5–43°C), as described above, became known. Nowadays, mild HT has become the standard in modern clinical trials and daily clinical usage (5). Modern HT technology has been developed and optimized for minimal hot spot occurrence as a main focus (51, 52). As a consequence, therapy-limitation due to focal hot spots has not been an issue in many recent HT trials (62–64).

### Temperature Control

As important as heat generation, measurement of the actual tissue temperature distribution is crucial for effective heating of tumors. A homogenous temperature distribution is necessary for optimal treatment effects. Local dose-limiting hotspots have to be avoided. Originally, temperature assessment was restricted to single-point measurements. It can be performed either invasively by insertion of intratumoral catheters or, as applicable in tumors with close proximity to natural cavities such as rectal, cervical, vaginal, urethral or vesical tumors, equally efficient by endoluminal catheters. Insertion of catheters inherits the risk of complications such as pain, inflammation, or abscess formation (65). Thus, the latter option should be used if possible. In superficial tumors, surface skin measurements by contact electrodes constitute a further alternative. In addition, by using infrared thermography cameras, two-dimensional data can be obtained for superficial tumors even though calibration with contact electrodes is necessary for absolute temperature assessment (56). A promising method for deep temperature monitoring is MRI-guided thermometry capable of measuring three-dimensional temperature distributions non-invasively. Temperature can be measured by exploiting either T1w-imaging, diffusion weighted imaging or proton resonance frequency shift-imaging (66). Proton resonance frequency shift-imaging appears to be the most accurate method in the clinical setting. By combining HT with online MRI thermometry, direct changes of temperature delivery can be performed to optimize temperature distribution and suppress hot spots (67). To this end, an adaptive iterative algorithm has been developed (68). First studies have confirmed its applicability in the clinical setting (69).

### Interval of Administration

In general, a dose–effect relationship of HT has been shown (70). The interval of administration of HT relative to RT has great influence on its effectiveness. The actual effect is quantified with the thermal enhancement ratio (TER) defined as the ratio of the respective radiation doses of RT alone divided by the RT + HT dose necessary to receive equal survival curves (4). However, biological aspects of HT react differently to the extent of heating sequence of HT. In the clinical setting, the maximal achievable TER should be combined with the most limited TER for healthy tissue to retain a tumor-specific effect reducing toxicity.

The inhibition of DNA repair has its highest effect when HT is given simultaneously to RT. The effect declines with the end of DNA repair mechanisms approximately 4 h after RT. However, inhibition of DNA repair mechanisms is not tumor specific since it is also present in normal tissues (71, 72). In contrast, direct cell killing is specific to malignant tissue at target temperatures. The respective TER is estimated at 1.5 deriving most likely from direct radiation-independent cell damage to radioresistant hypoxic cells (73). To summarize, optimal effects can be achieved by simultaneous RTHT treatment with no tumor-directed specificity. Treatment selectiveness would completely depend on accuracy of radiation delivery. In the time frame of 1–4 h before or after radiation, a maximal selective TER can be achieved by adding up DNA repair inhibition and direct cell damage. For optimization of the oxygenation-effect, RT should be applied shortly after HT (72). So far, the shortest interval between HT and RT has been described for wIRA followed by RT (56).

When considering time schedules and fractionation schemes, one should also take into account two phenomena termed cellular and vascular thermotolerance. The underlying mechanisms remain incompletely understood (74–77). For this reason, detailed description of these phenomena is not performed in the context of this review. Most probably, thermotolerance in currently used clinical HT schedules (i.e., once or twice/week) seems to play no limiting role.

## Clinical Evidence of Combined Thermoradiotherapy

In the following, key studies providing evidence for a clinical benefit of a combined treatment with HT and RT is presented, sorted by tumor entity and the extent of existing evidence (see Tables 1–3 for a detailed overview). In the following paragraph, existing clinical evidence of combined HT with CT is summarized (see Table 4).

**Table 1:** Summary of cited meta-analyses and randomized trials for breast cancer and cervical cancer.

Reference	Year	Entity	Study type	Treatment arms	RT/CT schedule	P #	HT frequency	Outcome
**Breast cancer**								
Vernon et al. (60)	1995	Inoperable primary/recurrent breast cancer	MA (5 r trials)	RTHT	Various: effective RT dose 39.8–60 Gy	171	mostly 1x/w	CR 59%,
				RT		135	-	41%, OR 2.3, *p* < 0,001
Datta et al. (79)^a^	2016	Locoregional recurrence	MA (24 1a.s.) 8 (5 r 2a.s.)	RTHT	Various: 24–60 à 1.8–4 Gy	1,483	1–5x/w	Single arm: CR 63.4%
					m: 38.2	309	(m: 2)	Two arm: CR 60.2%
				RT		318	–	CR: 38.1%, OR 2.6, *p* < 0.001
				reRTHT^b^	m: 36.7 à m: 2.7 Gy^b^	779^b^	1–5x/w^b^	CR 66.4%^b^
**Cervical cancer**								
Lutgens et al. (82)	2010	Locally advanced cervix carcinoma	Cochrane MA: 6 r 2a.s.	RTHT	Various concepts: EBRT (40–70 Gy) ±BT (16–50 Gy)	135	1–3x/w	CR HR 0.56, *p* < 0.001
								LR HR 0.48, *p* < 0.001
								OS HR 0.67, *p* < 0.05
				RT		132	–	
Datta et al. (84)	2016	Locally advanced cervix carcinoma	Network MA: 6 r 2a.s.	RTHT	Various concepts: EBRT (40–70 Gy) ±BT (16–60 Gy)	170	1–3x/w	CR: vs. HT: OR 2.85, s
				RTCT	+Cisplatin	281	–	
				RTHTCT	+Cisplatin	231	2x/w	CR: vs. HT: OR 4.52, s vs. HTCT: OR 2.91, s
								PA: vs. RT: OR 5.57, s vs. CTRT: OR 2.65, s
				RT		125	–	
Lutgens et al. (86)	2016	Locally advanced cervix carcinoma	r pIII	RTCT	EBRT 50 Gy + BT 21/29 Gy +Cisplatin	43	–	EFS: HR 1.15, ns
								OS: HR 1.04, ns
								PRFS: HR 0.94, ns
				RTHT		44	1x/w	

*RT, CT, and HT schemes, *p*-value or significance status (s/ns) are described as mentioned in the original publications*.

*a.s., arm study; BT, brachytherapy; CT, chemotherapy; CR, complete response; EFS, event-free survival; HR, hazard ratio; HT, hyperthermia; m, mean; ns, not significant; LR, local recurrence; MA, meta-analysis; OS, overall survival; OR, odds ratio; PRFS, pelvic recurrent-free survival; P #, patient number; p, phase; PA, patients alive; w, week; r, randomized; RT, radiotherapy; s, significant; RTHT, thermoradiotherapy; RTCT, chemoradiotherapy; EBRT, external beam therapy; à, with a single dose of; x/w, times per week*.

*^a^Including 4 two-arm studies reported in the study by Vernon et al*.

*^b^Subgroup analysis*.

**Table 2:** Summary of cited meta-analyses and randomized trials for head and neck cancer and rectal cancer.

Reference	Year	Entity	Study type	Treatment arms	RT/CT schedule	P #	HT frequency	Outcome
**Head and neck cancers**								
Datta et al. (87)	2015	Head and neck cancers	MA (5 r 2a.s., 1 nr 2a.s.)	RT	32–80 à 1.8–2 Gy	232	–	CR: 39.6%
				RTHT		219	1–2x/w	CR: 62.5%, OR 2.92, *p* = 0.001, RR 1.61, *p* < 0.001
Kang et al. (88)	2013	Nasopharyngeal cancer (N2/3)	r pIII	RTCT	50/78 à 2 Gy +cisplatin	78	–	CR: 62.8%
								5y-DFS: 20.5%
								5y-OS: 50%
				RTCTHT	+cisplatin	76	3 groups: 0.5–2x/w	CT: 81.6%, *p* < 0.05
								5y-DFS: 51.3%, *p* < 0.05
								5y-OS:68.4%, *p* < 0.05
Hua et al. (89)	2011	Nasopharyngeal cancer	r pIII	RT(CT)	50/60/70 Gy ± BT: (15–20 Gy) ±(T3/4) cisplatin/5FU	90	–	CR: 81.1%
								5y-DFS: 63.1%
								5y-OS: 70.3%
				RT(CT)HT	+(T3/4) cisplatin/5FU	90	2x/w	CR: 95.6%, *p* = 0.003
								5y-DFS: 72.7%, *p* = 0.039
								5y-OS: 78.2%, *p* = 0.14
Zhao et al. (90)	2014	Nasopharyngeal cancer	r pIII	RTCT	50/70–74 Gy +cisplatin/paclitaxel	40	–	3y-OS: 53.5%
								mPFS: 37.5
				RTCTHT	+cisplatin/paclitaxel	43	3x/w	Better quality of life
								3y-OS: 73%, *p* = 0.041
								mPFS: 48 mo, *p* = 0.05
**Rectal cancer**								
De Haas-Kock et al. (93)	2009	Locally advanced rectal cancer	MA (6 r 2a.s.)	RT	40–50 Gy	258	–	
				RTHT		262	1–5x/w	2y-OS: HR 2.06, *p* = 0.001
								CR: RR: 2.81, *p* = 0.01

*RT, CT, and HT schemes, p-value are described as mentioned in the original publications*.

*a.s., arm study; BT, brachytherapy; CT, chemotherapy; CR, complete response; DFS, disease-free survival; HR, hazard ratio; HT, hyperthermia; m, mean; mo, months; nr, non-randomized; MA, meta–analysis; OS, overall survival; OR, odds ratio; P #, patient number; p, phase; w, week; PFS, progression-free survival; r, randomized; RR, relative risk; RT, radiotherapy; y, years; RTHT, thermoradiotherapy; RTCT, chemoradiotherapy; à, with a single dose of; x/w, times per week*.

**Table 3:** Summary of cited randomized trials for bladder cancer, melanoma, NSCLC, glioblastoma, and sarcoma.

Reference	Year	Entity	Study type	Treatment arms	RT/CT schedule	P #	HT frequency	Outcome
**Bladder cancer**								
Matsui et al. (99)	1991	Bladder cancer	r pII	RT	50–70 à 2 Gy	16	–	Response: 56%
				RTHT	40 à 2 Gy	38	2x/w	Response: 84%, *p* < 0.001
van der Zee et al. (61)^a^	2000	Bladder cancer	r pIII	RT	40/66–70 à 2 Gy	38	–	CR: 51%
								3y-OS: 22%
								3y-LC: 33%
				RTHT		52	1x/w	CR: 73%, *p* = 0.01
								3y-OS: 28%, ns
								3y-LC: 42%, ns
**Melanoma**								
Overgaard et al. (101)	1995	recurrent or metastatic malignant melanoma	r pIII	RT	24–27 à 8–9 Gy - 4-day interval	34	–	CR: 35%
								2y-LC: 28%
				RTHT		34	After each RT	CR: 62%, *p* < 0.05
								2y-LC: 46%, *p* = 0.008
**NSCLC**								
Mitsumori et al. (102)	2007	NSCLC (St. II–III)	r pIII	RT	40/66–70 à 2 Gy	40	–	1y-PFS: 29%
								OS: 38.1%
				RTHT	20.0–76.0 Gy	40	1x/w	1y-PFS: 69%, *p* < 0.036
								OS:43%, *p* = 0.868
**Glioblastoma**								
Sneed et al. (112)	1998	Glioblastoma	r pII/III	RTHT	59,4 à 1,8 Gy + BT 60 Gy (0.4–0.6 Gy/h)	40	1x before + after BT	2y survival: 31%
								TTP: 49 mo
**Sarcoma**								
Leopold et al. (116)	1989	STS	r pII	RTHT	50/50,4 à 2/1,8 Gy	8	1x/w	Severe histopathologic changes 9/9
				RTHT		9	2x/w	Severe histopathologic changes 3/8

*RT, CT, and HT schemes, *p*-value or significance status (s/ns) are described as mentioned in the original publications*.

*a.s., arm study; BT, brachytherapy; CT, chemotherapy; CR, complete response; HT, hyperthermia; LC, local control; m, mean; mo, months; ns, not significant; OS, overall survival; P #, patient number; p, phase; w, week; PFS, progression-free survival; r, randomized; RT, radiotherapy; RTHT, thermoradiotherapy; NSCLC, non-small cell lung cancer; TTP, time to progression; STS, soft tissue sarcomas; à, with a single dose of; x/w, times per week. ^a^As part of the Dutch hyperthermia trial*.

**Table 4:** Cited studies for RTHT indications with limited data.

Reference	Year	Entity	Study type	P #	RT/CT schedule	Treatment arms	HT frequency	Outcome
**Indications with limited data**								
Milani et al. (125)	2008	Preirradiated painful recurrent rectal cancer	nr pII	24	30.0–45.0 à 1,8 Gy + 5FU	RTHTCT	2x/w	Pain relief: 70%
								Local PFS: 15 mo
Kalapurakal et al. (127)	2003	Locally advanced or recurrent prostate cancer	nr pI/II	13	m: 39.6 Gy for ReRT	ReRTHT	2x/w	25% grade IV toxicities
					m: 66.6 Gy for RT	RTHT		Symptom relief: 100%
								OR 46%, RR 54%
Maluta et al. (128)	2011	Primary or recurrent locally advanced pancreatic cancer	nr pII	40	30–66 Gy	RTHTCT	2x/w	OS: 15 mo
					gemcitabine ± oxalilatin, cisplatin, 5FU			
				28		RTCT	2x/w	OS: 11 mo, *p* = 0.025
Dong and Wu (129)	2016	Hepatocellular carcinoma	r pII	40	Not specified	RTHT	2x/w	1y-recurrence: 10%
								1y-mortality: 12.5%
				40		RT	2x/w	1y-recurrence: 15%, *p* < 0.001
								1y-mortality: 20%, *p* < 0.001
Yu et al. (130)	2016	Chemorefractory liver metastasis of colorectal cancer	nr pII	10	whole liver RT: 21 à 3 Gy	RTHT	2x/w	Pain relief 30%
								PR 30%, stable disease 50%
Aktas et al. (131)	2007	Primary vaginal cancer	nr pII	7	48 à 2 Gy + BT (17 à 8,5 Gy)	RTHT	Not specified	Tumor size > 4 cm
								5y-OS: 68%
				32		RT(CT)	–	Tumor size < 4cm
								5y-OS: 57%, ns
Jones et al. (64)	2005	Superficial tumors: breast cancer, head and neck, melanoma	r pIII	56	m: 50 Gy	RTHT		CR: 66.1%
				52	m: 55 Gy	RT	–	CR: 42.3%, OR 2.7, *p* = 0.02

*RT, CT, and HT schemes, *p*-value or significance status (s/ns) are described as mentioned in the original publications*.

*CR, complete response; PR, partial response; BT, brachytherapy; CT, chemotherapy; HT, hyperthermia; m, mean; mo, months; nr, non-randomized; ns, not significant; OS, overall survival; OR, odds ratio; P #, patient number; p, phase; w, week; PR, partial response; PFS, progression-free survival; r, randomized; RT, radiotherapy; s, significant; RTHT, thermoradiotherapy; RTCT, chemoradiotherapy; à, with a single dose of; x/w, times per week*.

### Breast Cancer

Breast cancer constitutes the most widely investigated malignant entity. Vernon et al. published the results of five randomized trials conducted between 1981 and 1991 that were combined due to insufficient patient accrual. The pooled analysis of 306 patients with inoperable primary or recurrent disease yielded a significantly better complete response (CR) (RTHT: 59%, RT alone: 41%, odds ratio (OR): 2.3, *p* < 0.001) but no survival benefit. However, 50% of patients had metastases at the time of randomization. The effect was most prominent in preirradiated recurrent lesions. Skin toxicities such as blisters, ulceration, and necrosis were higher in the HT group, however with low impact on the patients well-being and generally treatable with conservative measures (60).

In cases of locoregional recurrence, breast surgery is recommended if possible. However, radical resection only appears to be feasible in 65% of patients and may cause significant treatment-related morbidity (78). Thus, RT constitutes a significant alternative, and depending on the time interval between first and second RT and other pretreatment characteristics offers substantial clinical benefit. Since nowadays most patients receive RT in the primary situation, HT may help to enhance the effects of reirradiation in the recurrence scenario, especially since in some clinical situations only reduced radiation doses may be prescribed. A recent meta-analysis by Datta et al. included 8 two-arm studies and 24 single-arm studies involving 2,110 patients with locoregional recurrent disease (79). CR was similar between one-arm studies (CR: 63.4%) and two-arm studies as well as significantly higher compared to RT alone (RTHT: 60.2%, RT: 38.1%, OR 2.6, *p* < 0.001). In preirradiated patients (779 patients), a CR of 66.4% was achieved (mean re-RT dose: 36.7 Gy). Treatment-related toxicity was overall not increased with mean acute grade 3/4 toxicities of 14.4%. Among the analyzed studies, there was great heterogeneity in RT dose, HT fraction schedules, total number of HT fractions, HT duration, or average achieved temperatures. However, no prognostic treatment variables could be identified in a subgroup analysis and meta-regression. Similar results were recently published by Linthorst et al. In a retrospective study encompassing 248 patients with unresectable recurrences, reirradiation + HT yielded a CR of 70% (80). In resectable cases, a regimen including surgery, RT, HT, and partly CT or hormone therapy, a local control (LC) rate of 78% after 5 years was achieved (81). Notter et al. have treated patients with locally recurrent breast cancer with RTHT using a hypofractionation scheme of 5 × 4 Gy with one fraction per week. A wIRA system was used for superficial HT. A CR rate of 61% was achieved without any treatment-related toxicities (56). In summary, there is sustained evidence demonstrating a value of adjunct HT in locoregional, recurrent breast cancer as definitive or adjuvant treatment.

### Cervical Cancer

Several randomized trials have been conducted to test HT in combination with RT. A Cochrane database meta-analysis was performed analyzing six trials involving a total of 487 patients (82). The studies included mostly patients with locally advanced disease (74% FIGO stage IIIB). Except in one study, RT was delivered as a combination of external beam therapy (EBRT) and brachytherapy (BT). The pooled data analysis showed a significantly higher CR [CR relative risk (RR): 0.56, *p* < 0.001] and OS [hazard ratio (HR): 0.67, *p* = 0.05] in favor of RTHT as well as a reduced local recurrence rate (HR: 0.48, *p* < 0.001). No difference was found regarding acute and late toxicity rates. The authors criticized a lack of uniformity among the trials concerning HT delivery, HT schedules, as well as RT treatment protocols, RT dose, and RT techniques. Therefore, the authors conclude that the results do not suffice for a definitive recommendation to apply HT along standard treatment regimen. The included Dutch deep HT trial was updated for long-term results after 12 years of follow-up showing sustained improved LC (RT: 37%, RTHT: 57%, *p* = 0.01) and survival (RT: 20%, RTHT: 37%, *p* = 0.03) (83). All studies have in common that concurrent CT was not included in the treatment regimen.

In recent years, many trials have discussed the value of the combined treatment regimes such as RTHT, RTCT, or trimodal RTHTCT. A recently published meta-analysis has revised all relevant publications including 23 articles with a total patient number of 1,160 (84). In a network meta-analysis (NMA), all four possible treatment modalities (RT, RTHT, RTCT, RTHTCT) were compared. All studies, but one, included patients with locally advanced disease. RT always comprised EBRT + BT. The comparison of RTHT and RT yielded similar results as the previously described Cochrane analysis. The same six trials with minor updates were included. For the direct comparison of RTHTCT and RTCT, only one study including 68 patients was available (85). It showed a significant better CR for the trimodal treatment arm (RTCT: 46.7%, RTHTCT: 83.3%, risk difference 36.7%, *p* = 0.0001). No significantly increased grade 3/4 toxicities were found. The NMA showed a significant advantage of RTHTCT over all other treatment combinations in direct and indirect comparisons as well as in the SUCRA value-based ranking for CR and patients alive. RTCT and RTHT had similar performance even though RTHT had a small advantage over RTCT regarding CR. In coherence, a recent randomized phase III trial, which was closed early due to poor accrual with only 87 of 376 planned patients, did not show any significant difference in event-free survival and pelvic recurrence-free survival between RTHT and RTCT (86). In summary, by adding radiosensitizing treatments such as CT or HT to RT, better treatment outcomes are achievable. In locally advanced cervical cancers, RTHT appears to be a valuable substitute for RTCT if CT is not applicable. By combining all three modalities, the best treatment effect may be possible. Additional phase III trials are necessary directly comparing RTHT, RTCT, and RTHTCT for optimal treatment stratification.

### Head and Neck Cancers

To evaluate the effect of HT in head and neck carcinomas, a meta-analysis was recently performed including six 2-armed studies encompassing 451 patients. Five of the six studies were randomized trials. The CR rate appeared to be significantly better in patients treated with combined RTHT compared to RT alone (RT alone: 39.6%, RTHT: 62.5%, OR 2.92, *p* < 0.0001). Acute and late grade 3/4 toxicities were not significantly different (87). One study included exclusively nasopharyngeal carcinomas, whereas all other studies considered all cancer sites of the head and neck. However, all studies involving surgery or concurrent CT were excluded.

Three other randomized trials with a total of 417 patients recently analyzed the effects of trimodal treatment combing RT, CT, and HT in patients with nasopharyngeal carcinomas (88–90). Two studies reporting CR showed a significant advantgae for RTHTCT treatment. The same patients had an increased OS in two of the studies. Progression-free survival (PFS) or disease-free survival (DFS) was significantly better in the RTHTCT group in all three studies. Patients with higher tumor temperatures and higher HT fraction numbers showed a better outcome (88). In all three studies, no difference in treatment-related toxicity has been described. Patients receiving HT showed even better quality of life scores after completion of therapy (90). These studies demonstrate that trimodal therapy including RT, CT with different agents, and HT constitute an effective and safe treatment alternative. To our knowledge, no other randomized studies have been published in other head and neck sites investigating trimodal therapy. As shown in other malignancies, re-treatment may constitute a further clinical situation in which HT may be a valuable treatment option. In a small cohort receiving reirradiations combined with HT, a CR of 46% was achieved showing feasibility of such an approach (91). To conclude, HT constitutes a valuable treatment option in cancers of the head and neck. However, due to relatively high perfusion rates and fast adaptation to local temperature changes, HT delivery appears to be especially challenging. By using a site-tailored radiative heating device, treatment outcomes may be better in the future (92). HT could be used in multimodal treatment schemes further improving treatment outcome. Alternatively, it may constitute a toxicity-sparing alternative for concurrent CT in elderly or multimorbid patients. Further clinical studies are necessary to evaluate the true clinical value.

### Rectal Cancer

In 2009, a Cochrane analysis of six phase II and III randomized-controlled trials including 520 patients with locally advanced rectal carcinomas was performed. Patients received neoadjuvant RT with or without HT. Increased CR (RR 2.81, *p* = 0.01) as well as increased OS at 2y follow-up (HR 2.06, *p* = 0.001) could be observed. The survival benefit, however, could not be measured for any later time point. No difference in acute toxicity was found in the two studies reporting on this side effect (93). A positive impact on pathologic complete response (pCR) could as well be shown in a retrospective study of 106 patients. Sphincter-sparing surgery was higher for tumors in close proximity to the anal verge (94). In a further retrospective study encompassing 235 patients, HT appeared to confer better downstaging of the primary tumor and involved lymph nodes (95). Two small studies evaluated hypofractionated RTHTCT schemes showing principal efficacy and safety (96, 97). Additional HT appears to be well tolerated without increased impairment of quality of life (98). To conclude, the Cochrane analysis demonstrated a fundamental possibility of increased response by applying adjunct HT. However, further randomized prospective trials are necessary to evaluate the true value of neoadjuvant RTHTCT as well as of treatment of recurrent disease.

### Bladder Cancer

For the treatment of bladder carcinomas, HT has been predominantly applied in combination with intravesical CT. However, a few trials have evaluated RTHT. In an early study, 56 patients with bladder carcinomas were treated with intravesical CT (bleomycin) simultaneously to RTHT with reduced total dose (40 Gy) or RT alone with higher dose prescription (50–70 Gy). HT was delivered by intravesical infusion of warmed saline solution containing bleomycin. The RTHT group had a higher response rates (RTHT: 84%, RT: 56%, *p* < 0.001) with decreased toxicity rates (less bladder capacity reduction) (99). In a different approach, 49 patients with nodal-negative disease of all T stages were treated with a hypofractionated RT scheme (24 Gy, 4 Gy/fraction) with or without HT. The HT group was split into a high (Tmean > 41.5°C) and a low temperature cohort (Tmean < 41.5°C). The high temperature cohort showed significantly better downstaging compared to both other groups indicating the importance of adequate temperature delivery (100). In a more recent German trial, high-risk T1 and T2 cancers were treated with transurethral resection followed by RTHTCT (50.4 Gy + 5.4–9 Gy; cisplatin and 5FU). At six weeks follow-up, a pCR of 96% was achieved. After a median follow-up of 34 months, OS was 89% with 80% of the patients being satisfied with their bladder function (63). The Dutch deep HT trial also included bladder carcinomas besides cervical and rectal carcinomas. In a randomized protocol, 101 patients with T2–T4 N0 M0 bladder carcinoma were treated with either RT or RTHT. RTHT yielded a significantly better CR (RTHT: 73%, RT: 51%, *p* = 0.01). However at 3y, LC and OS were not significantly different. There was no difference in toxicity (61). Taken together, some studies exist demonstrating a clinical benefit for adjunct HT with no additional toxicity. However, the patient cohorts in the different studies appeared to be quite heterogeneous by mixing locally restricted and advanced tumors. The treatment regimens used differed among studies impairing adequate comparisons. Randomized studies are necessary with clearly defined risk profiles and adequate direct comparisons with guideline-based treatment regiments.

### Melanoma

One multicentric randomized trial analyzed the benefit of adjunct HT in melanomas treated with RT. Patients either received RT (24 Gy or 27 Gy in three fractions) alone or with HT (43°C for 60 min). There was no significant difference in toxicity. CR (RT alone: 35%, RTHT: 62%, *p* < 0.05) and LC after 2y were significantly increased (RT alone: 28%, RTHT: 46%, *p* = 0.008) (101). As these results are very promising, more randomized trials would help to establish a distinct role for RTHT in the treatment of melanomas, for example in combination with less hypofractionated treatment schemes.

### Non-Small Cell Lung Cancer (NSCLC)

Only few studies have investigated the role of HT for the treatment of NSCLC. A multi-institutional prospective randomized trial investigated the role of HT in addition to primary RT for locally advanced NSCLCs. No significant difference of OS, local response, or treatment-related toxicity could be observed. However, with a significantly higher 1y-PFS, a certain benefit was apparent (67.5%, 29%, *p* = 0.036) (102). In a small case–control study encompassing 13 patients with direct bone invasion treated with RTHT (60–70 Gy) showed a possibly high efficacy in LC and survival under this unfavorable condition (103). The benefit of HT in addition to reirradiation for recurrent NSCLC after primary RT was investigated in a small retrospective study involving 33 patients. Median doses used initially and for reirradiation were 70 and 50 Gy, respectively. Toxicity was moderate and limited to a maximum toxicity of grade 3 in 9% of patients. In patients with smaller tumors (<4 cm) and no distant metastases, long time survival was partly achieved (104). All three studies employed radiofrequency capacitive heating systems. So far, only limited evidence exists showing a true benefit of HT in NSCLC treatment. More studies are necessary to explore potential areas of application.

### Prostate Cancer

Feasibility of adjuvant HT treatment for prostate carcinomas has first been shown in two phase I/II studies involving locally advanced disease or recurrences after radical prostatectomy. Toxicity was limited to grades 2 and 3, respectively. Quality of life was not significantly changed by addition of HT to RT treatment (105). A HT-dependent burn occurred in one patient indicating critical temperature delivery (106, 107). Similar results were obtained in a larger phase II study involving 144 patients with high-risk disease (T2 + serum-PSA > 10 ng/ml or Gleason score ≥ 7) or locally advanced disease (T3/4) treated with RTHT and antihormonal therapy (108). A 5y-OS of 87% and 5y-biochemical PFS of 49% was observed with limited toxicity (maximum grade 2). Hurwitz et al. combined radiation with or without hormone deprivation therapy and transrectal ultrasound HT in locally advanced disease showing promising results with a 2y-DFS of 84% compared to historical 2y-DFS of 64% observed in patients in the 4-month androgen suppression cohort of the RTOG 92-02 trial (62). Currently, a phase II study examines the safety of combining HT and dose-escalated external salvage RT for recurrent prostate cancer (109). Another study is examining salvage BT combined with interstitial HT (110). In a retrospective analysis of 146 patients, no significant difference was found between patients receiving HT or not. The authors discussed that this might be due to insufficient heat delivery, since a significant difference was apparent for patients receiving a high thermal dose (111). To summarize, a set of phase II studies show promising results. However, randomized phase III trials are necessary to evaluate the actual value of adjuvant HT in the treatment of prostate carcinomas.

### Glioblastoma Multiforme (GBM)

In 1998, Sneed et al. investigated the impact of adjuvant interstitial HT after a BT boost for patients with newly diagnosed, supratentorial GBM smaller than 5 cm treated with postoperative RT and concomitant hydroxyurea. After patient exclusions, 68 patients were randomized to BT with or without HT (112). HT was administered 30 min before and after a BT boost *via* placement of helical-coil microwave antennas. The HT group showed significantly increased survival (2y-survival 31% vs. 15%, *p* = 0.02) and time to progression (TTP) (median TTP 49 vs. 33 months, *p* = 0.045); however, this treatment was accompanied by increased grade 3 toxicity rates. In recent years, efforts were made to optimize HT delivery by improving interstitial catheters or applying focused ultrasound (113, 114). No data exist so far validating these new techniques or the combination with temozolomide.

### Sarcomas

Randomized trials have shown a significant benefit of HT in addition to CT (115). However, evidence of combined RTHT in sarcoma treatment remains scarce. An early phase II study involving 17 soft tissue sarcomas (STS) patients neoadjuvant RTHT with twice weekly HT showed significantly more extensive changes in histopathological examinations than the once weekly HT group (116). In another study, 16 patients received irradiation with concurrent HT for the treatment of radiation-associated sarcomas (predominantly angiosarcoma) showing a total response rate of 75%. Toxicity was mild except one grade 4 adverse event (117). First clinical results show general feasibility of applying RTHT to sarcoma treatment. However, randomized trials are necessary to assess whether a similar benefit exists as it has been shown for neoadjuvant HTCT.

### Esophageal Cancer

Several phase II studies have investigated the feasibility of trimodal neoadjuvant RTHTCT treatment. Nakajima et al. treated 24 patients with neoadjuvant RTHTCT using docetaxel. A general response rate of 41.7% with a pCR rate of 17.6% was observed (118). Described toxicities were limited to grade 2 and grade 3. In a further study, 28 patients received RTHTCT with carboplatin and paclitaxel. R0 resection was possible in all patients with mild toxicity. Pathologic evaluation yielded a pCR rate of 19%. Treatment was tolerated well with mild toxicity rates (maximum grade 2) (119). A third study treated 35 patients with advanced disease with RTHTCT (bleomycin/cisplatin and 5FU) yielding a good CR rate of 33.3% (120). In addition, multiple retrospective analyses have shown a significantly increased survival benefit in favor of RTHTCT over RTCT (121–123). In summary, there is existing evidence promising a substantial clinical value of RTHTCT. However, randomized trials are necessary directly comparing RTHTCT to standardized treatment protocols involving RTCT.

### RTHT in Indications with Limited Data

Apart from the described entities, in multiple trials, RTHT has been applied to rather rare RT indications with only low levels of evidence (see Table 4 for a detailed summary of the cited trials).

A Dutch retrospective trial analyzed the efficacy of hypofractionated (28/32 Gy with a single dose of 4 Gy) reirradiation with concurrent HT for the palliation of painful unresectable recurrent rectal cancer with good to complete response in 72% of 47 patients (124). Similar results (70% pain relief) were reproduced in a prospective phase II trial by Milani et al. with normofractionated RTCTHT (125). Klaver et al. proposed a novel treatment strategy in a case series for locally advanced rectal cancer with concurrent peritoneal carcinomatosis by combing hyperthermic intraperitoneal chemotherapy with intraoperative RT showing general feasibility (126).

Thermoradiotherapy was evaluated for the palliation of symptomatic locally advanced or recurrent hormone-refractory prostate cancer in a small phase I/II study. All patients demonstrated partial response or complete response as well as complete symptom relief (127). However, two of eight preirradiated patients developed grade IV toxicities.

Primary or recurrent locally advanced pancreatic cancer was subject to an open-label study comparing RTCT with RTHTCT (gemcitabine ± 5FU/cisplatin/oxaliplatin) showing a significantly increased survival benefit without increased toxicity (mean OS: RTHTCT: 15 months, RTCT: 11 months, *p* = 0.025) (128). Moreover, the influence of adjunct HT for the treatment of liver lesions has been assessed. In a randomized Chinese trial of hepatocellular carcinoma patients, 1y-recurrence (RTHT: 10%, RT: 15%, *p* < 0.001) and mortality rates (RTHT: 12.5, RT: 20%, *p* < 0.001) were significantly lower for combined RTHT compared to RT alone (129). Chemorefractory colorectal cancer metastases were treated with whole-liver RT and concimittant HT showing partial response and pain relief in 30% of treated patients, respectively (130). Vaginal cancers have been chosen as target for HT in small prospective Dutch trial. Patients with vaginal carcinomas with a tumor size larger than 4 cm were treated with RTHT, whereas smaller tumors were primarily treated with RT showing no significant difference in 5y-survival (131).

In a more general approach, Jones et al. performed a randomized prospective trial pooling superficial tumors of various entities (breast carcinoma, melanoma, head and neck cancer, and others). Only tumors that appeared to be heatable on a pretest were randomized. Addition of HT to RT lead to significantly increased CR (RTHT: 66.1%, RT 42.3%, OR 1.7, *p* = 0.02). In coherence with many other studies, the highest difference was achieved in preirradiated patients (CR: RTHT: 68.2%, CR 23.5%) (64).

Despite the limited amount of evidence, substantial benefits of RTHT, especially for preirradiated, locally advanced and recurrent tumors, became apparent. Since there is a lack of randomized trials, definite recommendations for treatment cannot be made. In situations of recurrent or metastatic disease, RTHT may be justifiable as “individual treatment approach” on the basis of the existing evidence. In order to reach necessary patient number in potential future randomized trials, multiple entities with a similar condition (such as “recurrent,” “preirradiated,” or “locally advanced”) could be combined, following the approach of Jones et al. (64). In addition, HT centers should work more closely together for the establishment of multicenter trials capable of gathering critical patient numbers.

### Combined Thermochemotherapy (CTHT)

Thermochemotherapy has been evaluated in multiple clinical trials. In contrast to RTHT, CTHT is also being combined with WBHT. A limited number of phase II studies have shown feasibility of applying WBHT to CTHT treatment of various entities such as recurrent ovarian cancer, malignant pleural mesothelioma, metastatic STS, melanoma, and pretreated metastatic colorectal cancer (132–136). Up to date, no phase III trials exist. In contrast, regional HT has been evaluated in a large phase III trial (341 patients) of STS performed as joint effort by the European Organization for the Research and Treatment of Cancer and European Society for Hyperthermic Oncology. It showed a substantially and significantly improved local PFS, DFS, and OS after adding HT to EIA (etoposide, ifosfamide, doxorubicin) CT (HR for local progression/death: 0.58, *p* = 0.003, local 2y-PFS: HTCT: 76%, CT: 61%; 2y-DFS: CTHT: 58%, CT: 44%, *p* = 0.011; per-protocol OS: HR 0.66, *p* = 0.038) changing daily clinical practice in HT treatment centers (115). Further randomized trials have evaluated neoadjuvant CTHT in esophageal carcinoma (40 patients; histologic effectiveness: CTHT 58.3, CT: 14.3, *p* < 0.05) and adjuvant CTHT after transurethral resection of bladder carcinomas (83 patients; 10y-DFS: CTHT: 53%, CT: 15%, *p* < 0.001) demonstrating increased treatment efficacy (137, 138). A randomized trial with NSCLC showed a small benefit regarding “clinical benefit response” (80 patients, CTHT: 82.5%, CT: 47.5%, *p* < 0.05) (139). General feasibility of local CTHT has also been shown in other entities such as refractory or recurrent non-testicular germ cell carcinomas, recurrent or persistent ovarian cancer, breast carcinoma, or peritoneal carcinomatosis in several phase II studies (140–143). As alternative to regional HT, hyperthermic isolated limb perfusion has been established for the treatment of STS and unresectable melanomas showing favorable results (144, 145). In summary, the few existing randomized trials suggest substantial benefit by adding HT to CT. More randomized trials are necessary to broaden the spectrum of CTHT.

## Outlook

Review of the current literature has shown various retrospective and prospective trials exploring the value of adding HT to RT or RTCT regiments in multiple tumor entities. As in some entities, the real benefit of HT remains elusive, in other malignancies sustained evidence has been acquired. When considering the currently existing studies, a substantial part of evidence was gathered between the 1980s and early 2000s. Apart from technical aspects of HT, RT techniques have dramatically evolved since then. Nowadays, even better results in regard to treatment outcome as well as toxicity could be expected. Still, no widespread use of HT has been established in the last decades. Several reasons, such as reimbursement issues in certain countries, technical complexities, and challenges of homogenous heating and temperature monitoring, may have contributed to this fact. As more and more clinical trials are being published, the willingness/memorandum to make the effort of establishing HT in a rising number of institutions has increased.

The development of novel techniques with more exact heat delivery and temperature monitoring capacities may help to gain higher acceptance among physicians. HT may as well be further improved by better planning techniques. Mathematical modeling of the earlier mentioned HT effects on cell biology has paved the way to actual thermoradiotherapy planning (4). By integrating the biological HT effect into the LQ-model, a more exact RTHT treatment planning becomes possible. By adding online temperature control, as it can be achieved by MRI thermometry, temperature distribution could be optimized even further by real-time adjustments.

In the trials performed so far, HT delivery specifications were highly heterogeneous. HT frequency varied between once weekly to daily applications. Mean achieved temperature profiles differed vastly between 39 and 43°C. In trials using multiple HT specification, higher temperatures or HT frequencies were associated with better outcome (88, 100, 116). This underlines the necessity of optimizing HT schedules to optimize the treatment effect. To this end, larger randomized studies are necessary directly comparing distinct HT specifications.

Even though there are still a lot of open questions, basic research has revealed many ways of action of HT. Regarding the biological mechanisms of HT, combining HT with drugs exploiting underlying mechanisms may further increase radiosensitization. As an example, HT has been combined with antiangiogenesis agents. HT itself appears to directly impair angiogenesis at least in part by plasminogen activator inhibitor 1 induction (146). By combining HT with VEGFR2-inhibitor treatment, a synergistic antiangiogenesis effect *in vivo* has been shown to inhibit tumor growth (147). Regarding the immunogenic effects of HT, adjunct immunotherapy, such as checkpoint inhibition, constitutes a further interesting field of research. Before clinical trials can be designed, more basic research is necessary to evaluate the effects of thermo-immunotherapy in preclinical models.

All studies mentioned above have used HT in combination with photon-based irradiation. However, all over the world, an increasing number of particle beam facilities are being installed. Due to technical improvements, smaller and low-cost proton facilities may become available in the future possibly enabling a further widespread use. In contrast, facilities capable of delivering heavy ion-based irradiation with ^12^C remain scarce. In a brief summary, proton and ^12^C-ions share similar favorable dose distributions with low entry dose, a high dose in the “Bragg peak” followed by a more or less steep dose decline (148). In contrast to protons, ^12^C-ions inherit a significantly higher linear energy transfer and relative biological effectiveness (149). Several biological factors have been identified such as a low oxygen enhancement ratio (OER), less cell-cycle-dependent cell killing, inhibition of non-homologous DNA repair, cluster damages to the DNA, and more efficient cell killing of tumor stem cells (150). HT triggers killing of hypoxic, acidic as well as energy-deprived tumor cells, decreases OER, and confers direct killing of S-phased cells. Therefore, it has been proposed that combined therapy of proton irradiation with HT may have similar effectiveness as ^12^C beam therapy alone (151, 152). To the best of our knowledge, no data of the clinical use of simultaneous thermo-particle therapy has been published. The HYPROSAR phase I/II study is currently recruiting patients with unresectable STS at the Paul Scherrer Institut in Switzerland. Weekly HT is combined with proton beam therapy to achieve tumor downstaging with subsequent resection. There has only been one randomized trial treating 151 patients with uveal melanoma with or without adjuvant transpupillary thermotherapy months after the end of proton irradiation (153). Indeed, the rate of secondary enucleation was significant lower. Since the therapy was not conducted in direct temporal proximity, the abovementioned factors would not have taken effect. Hence, clinical trials are necessary to explore the benefit of combinational therapy. Direct comparison of thermo-particle therapy with ^12^C beam therapy should be performed in randomized trials.

Different technical solutions of external HT delivery have been discussed. Advances in the field of nanomedicine have introduced a novel approach for targeted HT by development of magnetic or superparamagnetic nanoparticles as recently reviewd by Datta et al. (154). Particles with cores of iron oxide or gold shells have already made their way into clinical phase I trials. Bergs et al. recently reviewed the existing particle constructs (155). Tumor-specific targeting might be achieved by passive accumulation in the tumor due to the aberrant vasculature with increased leakage and simultaneous impairment of lymphatic drainage (156). Alternatively, active targeting could be achieved by coating with tumor-specific antibodies or ligands (157). Heat can then be generated by applying external magnetic fields with rapid field alternations (158). In clinical phase I trials, dispersed nanoparticles were directly deposited at the tumor side either by percutaneous injection or intraoperatively. Only mild toxicities and quality of life impairments were observed. A maximum temperature of up to 55°C was achieved but target coverage remained insufficient (159–161). The advantage of nanoparticle-based HT may lead to a more selective heat delivery to the tumor with possibly higher temperatures and lower toxicity to adjacent normal tissues. By carrying chemotherapeutic agents, antibodies, or gene silencing RNA residues, nanoparticles may open completely new therapeutic opportunities (154). On the other side, tumor volume coverage is still far from optimal. Poorly perfused regions of tumors, in which HT has its greatest potential for radiosensitization, tend to “collect” lower particle numbers. Accumulation of particles in non-malignant tissues such as the reticuloendothelial system or by the glomerular filter of the kidney carries the risk of side effects (162). Microscopic disease, e.g., in lymphatic tissue may not be reached by sufficient high temperatures. Until safe appliance in the clinic becomes possible, more research is necessary to assess the biological risks and to optimize particle distribution and targeting. If these problems can be addressed, nanoparticles may be a valuable alternative to external HT.

Besides enhancement of RT effects, HT may also be used for radiation dose reduction. As described above, Notter et al. used a hypofractionation treatment scheme (5 × 4 Gy, one fraction per week) to treat patients with locally recurrent breast cancer. A similar CR rate of 61% was achieved compared to other studies using mean doses of 38.2 Gy (79). The relatively low dose enabled the authors to perform re-reirradiations. 17 patients with re-recurrences of lymphangiosis carcinomatosa received re-reirradiation following the same therapy scheme. A CR rate of 31% could be achieved (56).

Infections with human papilloma virus (HPV) are commonly found in carcinomas of the head and neck or cervix. HPV-positive tumors appear to be more radiosensitive than HPV-negative tumors. HT has been shown to trigger the degradation of the oncogenic HPV-derived E6 protein. As functional E6 binds and inactivates p53, HT may indirectly renew p53 activity favoring p53-dependent apoptosis. Thus, a combination of RT and HT may be particularly effective. To evaluate this effect, future trials should include HPV status for risk stratification (87). As a next step, clinical trials should test further RT dose de-escalation with concurrent HT in HPV-positive patients as it is already performed for RT alone (163, 164).

## Conclusion

There is abundant evidence demonstrating that HT constitutes a valuable supplement to currently performed RT or RTCT schemes improving tumor response in tumor entities such as head and neck or cervix. It also inherits great potential to enhance RT-based therapy of recurrent disease as it has been shown for breast cancer. Through continuous improvement of HT delivery, planning, and monitoring techniques, treatment effects may further improve. Novel combinations with targeted therapy agents, immunotherapy, nanomedicine, or particle therapy constitute promising fields of further research and interesting areas for future clinical application.

## Author Contributions

JP: reviewed literature and wrote the manuscript. PV: reviewed literature and wrote the manuscript. SC: reviewed literature and wrote the manuscript.

## Conflict of Interest Statement

The authors declare that the research was conducted in the absence of any commercial or financial relationships that could be construed as a potential conflict of interest.

## Abbreviations

BT, brachytherapy; CT, chemotherapy; DFS, disease-free survival; EBRT, external beam radiotherapy; HPV, human papilloma virus; HR, hazard ratio; HSP, heat shock protein; HT, hyperthermia; HTP, hyperthermia treatment planning; LC, local control; NK, natural killer; NMA, network meta-analysis; NSCLC, non-small cell lung cancer; OR, odds ratio; pCR, pathologic complete response; PFS, progression-free survival; RR, risk ratio; RT, radiation therapy; RTHT, thermoradiotherapy; RTCT, chemoradiotherapy; RTHTCT, thermochemoradiotherapy; SAR, specific absorption rate; TER, thermal enhancement ratio; TRTP, thermoradiotherapy planning; TTP, time to progression; WBHT, whole-body hyperthermia; wIRA, water-filtered infrared-A; y, year.
